# HIV-related restrictions on entry, residence and stay in the WHO European Region: a survey

**DOI:** 10.1186/1758-2652-13-2

**Published:** 2010-01-15

**Authors:** Jeffrey V Lazarus, Nadja Curth, Matthew Weait, Srdan Matic

**Affiliations:** 1 WHO Regional Office for Europe, Copenhagen, Denmark; 2 Department of Public Health, Copenhagen University, Copenhagen, Denmark; 3 Faculty of Lifelong Learning, Birkbeck College, London, UK

## Abstract

**Background:**

Back in 1987, the World Health Organization (WHO) concluded that the screening of international travellers was an ineffective way to prevent the spread of HIV. However, some countries still restrict the entrance and/or residency of foreigners with an HIV infection. HIV-related travel restrictions have serious implications for individual and public health, and violate internationally recognized human rights. In this study, we reviewed the current situation regarding HIV-related travel restrictions in the 53 countries of the WHO European Region.

**Methods:**

We retrieved the country-specific information chiefly from the Global Database on HIV Related Travel Restrictions at hivtravel.org. We simplified and standardized the database information to enable us to create an overview and compare countries. Where data was outdated, unclear or contradictory, we contacted WHO HIV focal points in the countries or appropriate non-governmental organizations. The United States Bureau of Consular Affairs website was also used to confirm and complement these data.

**Results:**

Our review revealed that there are no entry restrictions for people living with HIV in 51 countries in the WHO European Region. In 11 countries, foreigners living with HIV applying for long-term stays will not be granted a visa. These countries are: Andorra, Armenia, Cyprus (denies access for non-European Union citizens), Hungary, Kazakhstan, Moldova, the Russian Federation, Tajikistan, Turkmenistan, Ukraine and Uzbekistan. In Uzbekistan, an HIV-positive foreigner cannot even enter the country, and in Georgia, we were not able to determine whether there were any HIV-related travel restrictions due to a lack of information.

**Conclusions:**

In 32% of the countries in the European Region, either there are some kind of HIV-related travel restrictions or we were unable to determine if such restrictions are in force. Most of these countries defend restrictions as being justified by public health concerns. However, there is no evidence that denying HIV-positive foreigners access to a country is effective in protecting public health. Governments should revise legislation on HIV-related travel restrictions. In the meantime, a joint effort is needed to draw attention to the continuing discrimination and stigmatization of people living with HIV that takes place in those European Region countries where such laws and policies are still in force.

## Background

We read the article, "Fear of foreigners: HIV-related restrictions on entry, stay and residence"[1], in this journal with great interest. In their contribution to the debate over HIV-related travel restrictions, Amon and Todrys stress the urgency on this issue, which affects not only the lives of people living with HIV (PLHIV) all over the world, but also the wellbeing of the communities in which they live. HIV-related travel restrictions not only violate the fundamental rights of PLHIV, but they also impede HIV prevention, care and treatment efforts among all people.

The United Nations Human Rights Committee has stated, "Liberty of movement is an indispensable condition for the free development of a person" [2]. Earlier, the Office of the High Commissioner for Human Rights stated that:

The [International] Covenant [on Civil and Political Rights] does not recognize the right of aliens to enter or reside in the territory of a State party. It is in principle a matter for the State to decide who it will admit to its territory. However, in certain circumstances an alien may enjoy the protection of the Covenant even in relation to entry or residence, for example, when considerations of non-discrimination, prohibition of inhuman treatment and respect for family life arise [3].

Governments do, of course, have the right to control entry to their borders and have a certain margin of appreciation to justify differential treatment compatible with international human rights law. But the measures must pursue a legitimate aim and need to be proportional to the achievement of this aim [4].

Back in 1987, the World Health Organization (WHO) concluded that the screening of international travellers was an ineffective way to prevent the spread of HIV [5]. In 2002, Member States of the WHO European Region resolved "to develop a supportive social and legal environment for groups at risk, especially sex workers, and for people living with HIV/AIDS and to fight social and legal exclusion, including travel restrictions" [6].

Since then, travel restrictions connected with communicable diseases in general and HIV in particular have often been discussed [7-9], including recently in conjunction with the 2009 outbreak of influenza virus A (H1N1). Together with international organizations, such as the International AIDS Society (IAS) [10], the International Organization for Migration and the Joint United Nations Programme on HIV/AIDS (UNAIDS) [11], Amon and Todrys emphasize how HIV-related travel restrictions have serious implications for individual and public health and violate internationally recognized human rights.

This important discussion prompted us to review the current situation in the 53 countries of the WHO European Region, given that restrictions on entry, residence and stay affect a wide range of PLHIV, including not only students and employees, but also members of vulnerable groups, such as refugees, asylum seekers and other migrants.

## Methods

In this study, which we carried out in April and May 2009, our concern was to map formal entry and residence restrictions that required an HIV test or a medical certificate of HIV status. It should be noted that in practice, however, some of the countries did not apply the rules that were legally valid at this time. We also reviewed whether people can be denied entry when applying for long-term stay (but not residence) or be deported if authorities obtain evidence of HIV infection.

To obtain a valid, up-to-date overview of HIV-related travel restrictions in the European Region, we collected data from a variety of sources. We retrieved the information chiefly from the Global Database on HIV Related Travel Restrictions at hivtravel.org[12], an initiative of the German AIDS Federation, the European AIDS Treatment Group (EATG) and the IAS. The information in this database is based on replies to a structured self-administered questionnaire from German embassies abroad and foreign embassies in Germany between November 2007 and June 2008.

We simplified and standardized the database information to enable us to create an overview and compare countries. Where data was outdated, unclear or contradictory, we searched the websites of foreign ministries in the countries and contacted WHO HIV focal points in the countries or appropriate non-governmental organizations (NGOs), such as the Eurasian Harm Reduction Network and the Hungarian Civil Liberties Union.

We also used the United States Bureau of Consular Affairs website [13] to confirm and complement these data. Most of the information provided by the focal points and NGOs was clear, sufficient and based on national laws and regulations. However, in some instances, the information was vague, and several communications were sometimes necessary to clarify unresolved questions.

## Results

For 11 of the 53 countries (Armenia, Belarus, Bulgaria, Cyprus, Georgia, Hungary, Israel, Moldova, Tajikistan, Ukraine and Uzbekistan), publicly available information did not provide a sufficient or clear picture of HIV-related travel restrictions. In these cases, we contacted focal points and NGOs, receiving replies from every country except Israel.

**Figure 1:**
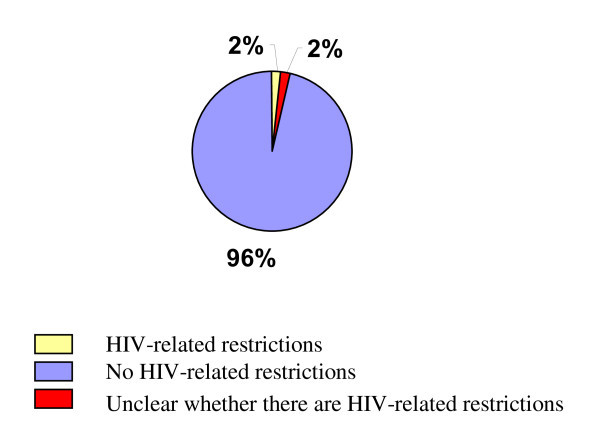
**Percentage of European Region countries with HIV-related entry restrictions**.

The resulting information and our initial review of the hivtravel.org database revealed that there are no entry restrictions for PLHIV in 51 countries (see Table S1, Additional file 1 and Figure 1). In Uzbekistan, however, the law mandates that visitors carry a certificate attesting that they are not infected with HIV. Foreigners from countries requiring visas to enter or stay in Uzbekistan will not be issued a visa to enter the country if they are found to be HIV positive. In Georgia, the situation for PLHIV wishing to enter the country is uncertain due to unclear information.

**Figure 2:**
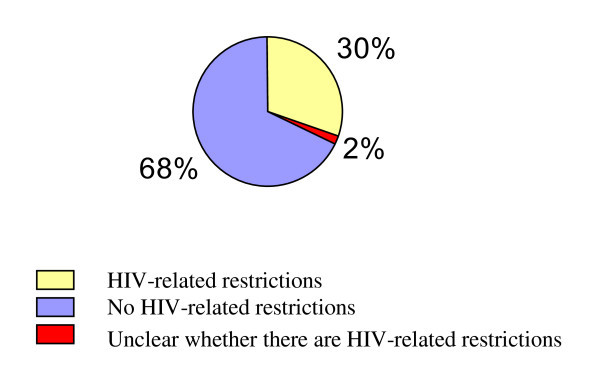
**Percentage of European Region countries with HIV-related residence restrictions**.

In 36 countries, there are also no HIV-related restrictions for long-term visits (see Figure 2). In Georgia, the policy on long-term visits is unclear. In eight countries (Belarus, Moldova, Poland, the Russian Federation, Tajikistan, Turkmenistan, Ukraine and Uzbekistan), an HIV test is required for all foreigners wishing to stay for more than three months. In three of these countries (Republic of Moldova, the Russian Federation and Turkmenistan), this requirement also applies to students and employees. In the Russian Federation, an HIV test is not required for citizens of countries in the Commonwealth of Independent States, who do not need visas for long-term stays.

**Figure 3:**
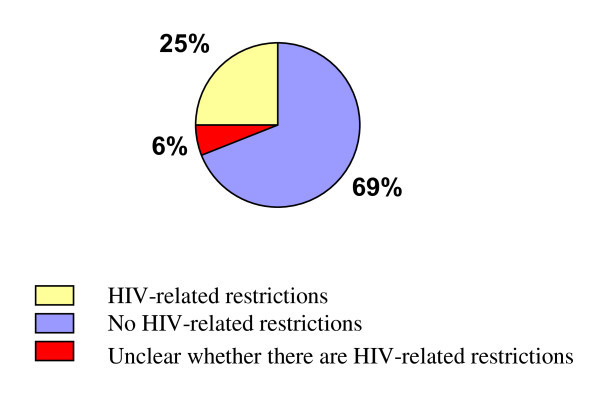
**Percentage of European Region countries with residence restrictions where a foreigner will not be granted a visa if found to be HIV positive**.

Andorra will not grant residency or work permits to PLHIV (See Figure 3). In Hungary, an HIV test is required of all foreigners wishing to stay for more than one year. In Kazakhstan, an HIV test is required for foreigners staying for more than 30 days. In Cyprus, people who are not citizens of the European Union must present an HIV test to apply for a work or study permit, which will be denied if the test is positive. In Slovakia, an HIV test is also required for foreigners applying for residence or a work permit. In the German state of Bavaria, an HIV test can be required for people staying for more than 180 days, while in the states of Saxony and New Brandenburg, there is mandatory HIV testing for asylum seekers.

In Armenia, the situation for long-term visitors is complex. A negative HIV certificate is required from all foreigners applying for visas. Until 14 July 2009, foreign PLHIV already in the country were subject to deportation. On that date, a new law came into force, specifying that foreigners would not be deported if found to be HIV positive. Yet a foreigner applying for a visa still has to present a negative HIV test. Armenia is working to change these regulations.

And finally, while we did not find sufficient information on requirements for long-term stays in Israel, there are indications that foreigners in general do not need to present a certificate of HIV status, although an HIV test is required for all migrant workers and for migrants from regions where HIV is endemic. However, it is not clear if a migrant can be denied access based on a positive HIV test.

Of the 17 countries requiring an HIV test or certificate for applying for long-term stays, 11 countries (69%) will deny a foreigner holding a positive HIV test entry into the country. In addition to Cyprus, which denies access to non-EU citizens, these countries are Andorra, Armenia, Hungary, Kazakhstan, Moldova, the Russian Federation (for citizens outside the Commonwealth of Independent States), Tajikistan, Turkmenistan, Ukraine and Uzbekistan.

## Discussion

Our research shows that only 36 out of 53 countries have no travel restrictions of any kind for PLHIV. This means that in 32% of the countries in the European Region, either there are some kind of HIV-related travel restrictions (as defined in this paper) or we were unable to determine if such restrictions are in force.

Although most countries with HIV-related travel restrictions defend them as being justified by public health concerns, the WHO Regional Office for Europe has explicitly rejected this claim [6]. Not only do HIV-related travel restrictions tend to be ineffective and lead to a false sense of protection - a country's nationals can just as easily contract the virus abroad and spread it at home, for example - but they also contribute to and reinforce the discrimination and stigmatization to which PLHIV are subjected. Further, people facing restrictive measures at entry may hide their status and avoid HIV testing and even health care services in general. Further, the European Union HIV/AIDS Civil Society Forum has called for the elimination of all HIV-related travel restrictions in Europe by 2010 [14].

The Office of the United Nations High Commissioner for Human Rights and UNAIDS, for example, have unequivocally stated that "any restrictions on these rights [to liberty of movement and choice of residence] based on suspected or real HIV status alone, including HIV screening of international travelers, are discriminatory and cannot be justified by public health concerns" [15] because while HIV is infectious, it cannot be transmitted through casual contact [16]. Those countries without HIV-related entry, stay, and residence restrictions have not reported any negative public health consequences [17].

Additional considerations arise with respect to travel within the 27 countries of the European Union because free movement of people within the EU is one of its founding principles, a principle acknowledged not only in its founding and subsequent treaties, but also in the European Convention of Human Rights. For example, Council Directive 2004/38/EC [18] states that:

Without prejudice to the provisions on travel documents applicable to national border controls, all Union citizens with a valid identity card or passport ... shall have the right to leave the territory of a Member State to travel to another Member State [Article 4.1].

And similarly:

Without prejudice to the provisions on travel documents applicable to national border controls, Member States shall grant Union citizens leave to enter their territory with a valid identity card or passport .... [Article 5.1].

The directive later notes that:

Subject to the provisions of this Chapter, Member States may restrict the freedom of movement and residence of Union citizens ... on grounds of public policy, public security or public health. These grounds shall not be invoked to serve economic ends [Article 27.1].

However, it goes on to place narrow limits on public health arguments for such restrictions:

The only diseases justifying measures restricting freedom of movement shall be the diseases with epidemic potential as defined by the relevant instruments of the World Health Organization and other infectious diseases or contagious parasitic diseases if they are the subject of protection provisions applying to nationals of the host Member State [Article 29.1].

For example, travel restrictions can be used to limit the spread of highly contagious diseases, such as cholera or acute respiratory syndrome (SARS), but such measures tend to be short-term and are most likely not very effective. Even in these cases, authorities must still consider human rights and the broad social, economic and public health consequences of initiating travel restrictions of any kind.

In general, WHO does not support travel restrictions in relation to communicable diseases, and the recent case of influenza A (H1N1) was no exception [19]. According to the International Health Regulations [20], a binding document signed by all WHO Member States, national health measures for travellers must not be more restrictive of international traffic, or more invasive or intrusive to the individual, than available alternatives that provide an appropriate level of health protection. If such measures are implemented, they should be justified by scientific principles, available scientific evidence or WHO advice. In the case of HIV, there is no evidence that denying HIV-positive foreigners access to a country is effective in protecting public health.

## Conclusion

In contrast to HIV, the highly contagious diseases that we have mentioned have short incubation periods and are transmitted through casual contact. While HIV transmission is mostly due to risk behaviours like sharing needles or unsafe sex, these diseases are transmitted much more readily, through droplets in the air or contaminated food or water. In the light of these differences, as well as the potential for discrimination and stigmatization, the public health justification for HIV-related travel restrictions is inadequate and even irrational.

## Competing interests

The authors declare that they have no competing interests.

## Authors' contributions

NC drafted the article based on an idea from JVL and SM. JVL fully revised the first draft and MW reviewed and added additional material. NC fact checked the changes. SM fully reviewed and edited the next draft. JVL and MW addressed the reviewer's comments. All authors read and approved the final manuscript.

## Supplementary Material

Additional file 1 **Table S1**. Overview of travel restrictions in the countries of the WHO European Region. Click here for file
