# Supplementary material for: HIV-related restrictions on entry, residence and stay in the WHO European Region: a survey
Source: J Int AIDS Soc. 2010 Jan 15;13:2. doi: 10.1186/1758-2652-13-2 (PMC2823611; doi:10.1186/1758-2652-13-2)
Supplement: Additional file 1 — Table S1. Overview of travel restrictions in the countries of the WHO European Region. [file 1758-2652-13-2-S1.DOC]

**Table S1. Overview of travel restrictions in the countries of the WHO European Region**

| **Country** | **Specific entry restrictions for PLWHIV** (HIV test or health certificate required at entry) | **Specific residence restrictions** (HIV test or HIV specific health certificate required when seeking residency or working permit) | **Deportation/denied visa application (long term stay) if HIV positive** |
| --- | --- | --- | --- |
| **Albania** | No | No | No |
| **Andorra** | No | Yes  – PLHIV cannot be granted residency or a working permit | Yes |
| **Armenia** | No | Yes | Yes (visa) No (deportation) |
| **Austria** | No | No | No |
| **Azerbaijan** | No | No | No |
| **Belarus** | No | Yes  – An HIV-test is required for people staying longer than three month | No |
| **Belgium** | No | No | No |
| **Bosnia and Herzegovina** | No | No | No |
| **Bulgaria** | No | No | No |
| **Croatia** | No | No | No |
| **Cyprus** | No | Yes  – Non-EU citizens that apply for residence in order to study or work must perform an HIV test | Yes  If the HIV test is positive, non-EU citizens will be denied access. |
| **Czech Republic** | No | No | No |
| **Denmark** | No | No | No |
| **Estonia** | No | No | No |
| **Finland** | No | No | No |
| **France** | No | No | No |
| **Georgia** | Unclear | Unclear | Unclear |
| **Germany** | No | Yes  – For long-term stays there are specific regulations in Bavaria (HIV testing for people staying more than 180 days can take place), Saxony and Brandenburg. | Yes  – **In Saxony there have been reports of cases of PLHIV being denied residency.** |
| **Greece** | No | No | No |
| **Hungary** | No | Yes  – An HIV test is required for foreigners wishing to stay more than one year. Some employers also request HIV tests. | Yes  – HIV status can be a reason for denying residency and deportation of HIV positive foreigners can take place |
| **Iceland** | No | No | No |
| **Ireland** | No | No | No |
| **Israel** | No | Yes  – An HIV test is required for documented migrants from endemic regions as well as migrant workers | Unclear |
| **Italy** | No | No | No |
| **Kazakhstan** | No | Yes  –When staying for more than 30 days | Yes |
| **Kyrgyzstan** | No | No | No |
| **Latvia** | No | No | No |
| **Lithuania** | No | No | No |
| **Luxembourg** | No | No | No |
| **Malta** | No | No | No |
| **Monaco** | No | No | No |
| **Montenegro** | No | No | No |
| **Netherlands** | No | No | No |
| **Norway** | No | No | No |
| **Poland** | No | Yes  – An HIV test is required for all foreigners wishing to stay in the country for more than three months.  There is mandatory HIV testing for pregnant women and babies that are suspected to be HIV infected. | No |
| **Portugal** | No | No | No |
| **Republic of Moldova** | No | Yes  – An HIV test is required for all foreigners wishing to stay in Moldova for more than three months (for residency, working or studying purposes). | Yes |
| **Romania** | No | No | No |
| **Russian Federation** | No | Yes  – An HIV test is required for foreigners wishing to stay for more than three months and students and employees  NoTE: This does not apply for citizens of non-visa CIS countries | Yes  – If an HIV infection is diagnosed, the foreigner has to leave the country within 3 months |
| **San Marino** | No | No | No |
| **Serbia** | No | No | No |
| **Slovakia** | No | Yes  – An HIV test is required for foreigners applying for a residence or working permit. | No |
| **Slovenia** | No | No | No |
| **Spain** | No | No | No |
| **Sweden** | No | No | No |
| **Switzerland** | No | No | No |
| **Tajikistan** | No | Yes  – An HIV negative test is required for all foreigners, wishing to stay in the country for more than three months | Yes  – Individuals with a positive HIV test will be deported |
| **The former Yugoslav Republic of Macedonia** | No | No | No |
| **Turkey** | No | No | No |
| **Turkmenistan** | No | Yes  – Foreigners wishing to stay for more than three months, students and applicants for work permits will have to present a HIV test | Yes  – Foreigners and stateless people refusing to do the testing or a preventive examination will be deported. Also if a person is discovered with an HIV infection the person can be denied a visa or can be deported. |
| **Ukraine** | No | Yes  – Foreigners wishing to stay for more than three months must present a negative HIV test. | Yes  – A HIV positive foreigner will not get permission to stay for more than three months |
| **United Kingdom of Great Britain and Northern Ireland** | No | No | No |
| **Uzbekistan** | Yes  – Uzbek law mandates that visitors carry a medical certificate attesting that they are not infected with HIV. | Yes  – An HIV negative test is required when wishing to stay for more than three months. | Yes  – All foreigners will be deported if they are found to be HIV positive |

***Sources:***

1. [www.hivtravel.org](http://www.hivtravel.org/).
2. US State Department web site: <http://travel.state.gov/travel/cis_pa_tw/cis/cis_1765.html>.
3. Personal correspondence with focal points in the country.
